# Prediction of stroke thrombolysis outcome using CT brain machine learning

**DOI:** 10.1016/j.nicl.2014.02.003

**Published:** 2014-03-30

**Authors:** Paul Bentley, Jeban Ganesalingam, Anoma Lalani Carlton Jones, Kate Mahady, Sarah Epton, Paul Rinne, Pankaj Sharma, Omid Halse, Amrish Mehta, Daniel Rueckert

**Affiliations:** Division of Brain Sciences, Imperial College London, Charing Cross Hospital Campus, Fulham Palace Rd., London W6 8RF, UK; Biomedical Image Analysis Group, Dept. of Computing, Imperial College London, South Kensington Campus, UK

**Keywords:** Stroke, Thrombolysis, Prediction, Machine learning, Imaging

## Abstract

A critical decision-step in the emergency treatment of ischemic stroke is whether or not to administer thrombolysis — a treatment that can result in good recovery, or deterioration due to symptomatic intracranial haemorrhage (SICH). Certain imaging features based upon early computerized tomography (CT), in combination with clinical variables, have been found to predict SICH, albeit with modest accuracy. In this proof-of-concept study, we determine whether machine learning of CT images can predict which patients receiving tPA will develop SICH as opposed to showing clinical improvement with no haemorrhage. Clinical records and CT brains of 116 acute ischemic stroke patients treated with intravenous thrombolysis were collected retrospectively (including 16 who developed SICH). The sample was split into training (n = 106) and test sets (n = 10), repeatedly for 1760 different combinations. CT brain images acted as inputs into a support vector machine (SVM), along with clinical severity. Performance of the SVM was compared with established prognostication tools (SEDAN and HAT scores; original, or after adaptation to our cohort). Predictive performance, assessed as area under receiver-operating-characteristic curve (AUC), of the SVM (0.744) compared favourably with that of prognostic scores (original and adapted versions: 0.626–0.720; p < 0.01). The SVM also identified 9 out of 16 SICHs, as opposed to 1–5 using prognostic scores, assuming a 10% SICH frequency (p < 0.001). In summary, machine learning methods applied to acute stroke CT images offer automation, and potentially improved performance, for prediction of SICH following thrombolysis. Larger-scale cohorts, and incorporation of advanced imaging, should be tested with such methods.

## Highlights

• Machine learning of acute stroke CTs may predict thrombolysis-associated haemorrhage.
• CT machine learning also circumvents high variability of radiologist interpretations.
• Favourable performance of CT machine learning reported here warrants further research.

## 1 Introduction

Intravenous thrombolysis (tPA) is the most efficacious treatment for acute ischemic stroke, but suffers a major complication rate of ~ 6% (Wardlaw et al., 2012), due to symptomatic intracranial haemorrhage (SICH). Whilst multiple factors have been associated with SICH (Whiteley et al., 2012), selection of patients on the basis of anyone of these, e.g. age or stroke severity, is not generally recommended (Leys and Cordonnier, 2012), since tPA appears to confer a net benefit across the range of all such baseline parameters (Sandercock et al., 2012). Recognising that single factors in isolation are poor predictors of SICH, several prognostic scoring systems have been developed (Lou et al., 2008; Strbian et al., 2012) which estimate a cumulative risk of SICH across a number of predictors, and provide thresholds above which tPA use is discouraged. However, these are far from perfect — with predictive performances, assessed by areas under receiver-operating characteristic curves (AUC), in large validation cohorts, of 60–70% (Mazya et al., 2013; Sung et al., 2013), partially explaining why such scores are not yet in routine clinical practice. Consequently, there is a compelling imperative to devise improved prognostic methods that can more accurately identify combinations of features that determine which patients will benefit from thrombolysis, versus be harmed by it.

An important set of predictors for SICH are radiological, based upon acute CT scan, and include: acute ischemia, vessel thrombosis and background white matter disease (Neumann-Haefelin et al., 2006; Whiteley et al., 2012). However, many such features – e.g. hypoattenuation of middle cerebral artery territory – are poorly discriminated by CT, resulting in inaccurate quantification, and significant inter-rater variability (Wardlaw and Mielke, 2005). Recently developed machine learning techniques (Orrù et al., 2012), that can classify images based upon features too subtle for the human eye, and recognise relevant patterns across a vast array of clinical/imaging inputs, may enhance our ability to predict SICH, and offer automation.

Here we test one machine learning method, previously successful in other neuroimaging diagnostic contexts (Gray et al., 2013; Orrù et al., 2012), for the ability to identify acute ischemic stroke patients at risk of tPA-associated SICH, using raw CT-images, as compared with radiologist-derived interpretations, in combination with clinical variables.

## 2 Methods

We retrospectively identified all 330 acute ischemic stroke patients treated with intravenous tPA in our hospital, for whom complete data existed. From this, we extracted all 16 patients who later developed SICH, defined here as any increase in National Institute for Health Stroke Scale (NIHSS) within 1 week – i.e. neurological deterioration – judged to be due to imaging-confirmed, intracranial haemorrhage. We chose a definition of SICH that incorporated any clinical deterioration, along with new haemorrhage (equivalent to that used in the National Institute of Neurological Disorders and Stroke (NINDS) trial), as opposed to more conservative definitions (Seet and Rabinstein, 2012), so as to maximise the number of cases, and since it is more likely that imaging features can be weighted so as to relate to haemorrhage potential per se, rather than haemorrhage resulting in deterioration above a certain clinical threshold.

Since this is a proof-of-concept study, and owing to the considerable resource intensity required by the multiple steps we performed on each subject (see later), we selected only 100 non-SICH subjects as controls. Downsampling of a group is also one method by which the problem of case–control imbalance, as faced here, can be lessened (Mazurowski et al., 2008). For this downsampling, we first excluded 28 non-SICH subjects (8%) who either incurred asymptomatic intracranial haemorrhage, or showed no NIHSS score improvement. The rationale for leaving out this group at an early stage of model development is that such subjects are less important to identify (the benefit of tPA in these subjects is less apparent than in controls whose NIHSS scores improved without bleeding), and because there is a higher chance that radiological features critical to SICH prediction would overlap with this group (especially patients who developed asymptomatic intracranial haemorrhage). Of the remaining 286 non-SICH patients, we selected a random but representative subsample of 100. This was achieved by repeatedly sampling 100 subjects without replacement from a uniform distribution over the original 286; comparing each with the remainder, using appropriate statistical tests for each clinical and radiological baseline characteristic; and selecting the first such subset for which statistical non-significance was found across all such comparisons. The characteristics of the selected and remainder non-SICH groups are shown in Table 1.

The following baseline variables (i.e. prior to tPA) were obtained from SICH and non-SICH groups: gender, age; treatment delay; NIHSS score; blood pressure; serum glucose; prothrombin time; platelet count; and prior anti-thrombotic therapy. Additionally, each baseline CT was interpreted by three independent neuroradiologists, for the following features: presence and extent of acute ischemia, hyperdense middle cerebral artery (MCA), and white-matter disease (Fazekas score) (Wahlund et al., 2001). Radiologists were blinded to outcome, but provided with clinical details (e.g. hemiparesis side) to mimic real-world judgements. Radiology results were combined by taking majority judgements or mean scores. Mean inter-rater variability (Cohen's κ) was 0.355 for acute ischemic changes, and 0.673 for hyperdense middle cerebral artery. Average time-to-scan was 9.4 min before quoted treatment delay (Pearson's r = 0.91; p < 0.01). TPA dosage was always 0.9 mg/kg, maximum 90 mg. The influence of each variable in determining SICH, versus not, was assessed with univariate logistic regression. Two validated SICH-prognostic tools – SEDAN (Strbian et al., 2012) and HAT (Lou et al., 2008) scores – that are composites of the above variables, were also compared between the two groups. Since we found in our cohort, that glucose and age non-significantly predicted SICH, and in order to match inputs between automated and standard approaches, we calculated ‘adapted’ SEDAN and HAT scores that included only their radiological and NIHSS components. The inter-rater reliability of SEDAN and HAT scores based upon the three radiology judgements was 0.639 and 0.624 respectively (linear-weighted Cohen's κ).

**Fig. 1:**
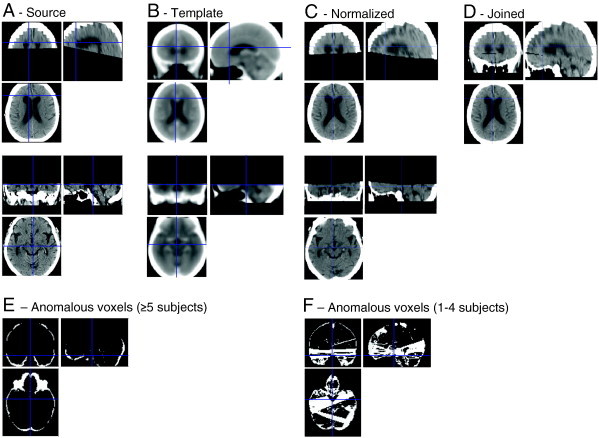
Example of CT normalisation pipeline in one subject. Source images were acquired in top and bottom sections (A) that were normalised respectively to whole-brain and bottom CT templates (B). The two normalised images (C) were joined (D) whereby voxels that were sampled in both images were averaged, and some voxels were sampled in neither (seen as a black ‘join’ anteriorly). The resultant images were inclusively masked by a brain template, but sometimes this included patients' cranium (revealed as anomalous Hounsfield Unit > 200): E shows where this occurred in ≥ 5 subjects, these voxels then being excluded. Most patients also showed a thin non-sampled join, although the location of this differed slightly across subjects. These and other anomalous voxels that occurred in < 5 subjects were replaced by their mean from the remaining set of subjects, and are identified in F.

Baseline CTs were acquired from one of two Siemens Definition AS + 128-slice scanners, in two sections covering upper two-thirds of the cerebrum (matrix size: 512 × 512 × (10 - 12); resolution: 0.4 × 0.4 × 9.0 mm) and brain base (512 × 512 ×  (18 - 24); 0.4 × 0.4 × 3.0 mm). Note that CT brains are often acquired in two parts in clinical practice, e.g. so as to enhance sensitivity to basal lesions. Using SPM8, upper and lower brain images were re-oriented, and spatially normalised to whole-brain and lower-brain CT templates respectively (derived from 30 healthy subjects with mean age of 65 (Rorden et al., 2012)), resulting in two images sized 79 × 95 × 68 (2 mm^3^ resolution) (Fig. 1A–C). The two images were joined in a common space, taking voxel averages where these overlapped (Fig. 1D). Resultant images were then masked inclusively for brain (without ventricles), resulting in 208,018 data-containing voxels per subject. From these, voxels were deleted where, in 5 or more subjects, these assumed anomalous values for brain (< 0, > 200 Hounsfield Units), resulting in 181,311 voxels per subject. Deleted anomalous values occurred mostly where patients' crania tended to overlap template-brain edges, due to imperfect spatial normalisation (Fig. 1E). For voxels where < 5 subjects showed anomalous values (most commonly due to non-sampled voxels at the join between the upper and lower sections) we replaced these values with their mean from the remaining sample. This way, we minimized the influence of rare anomalous values, whilst still enabling the rest of subjects to contribute data to these voxels (many of which were in potentially relevant areas around middle cerebral arteries, — see Fig. 1F). Images were then corrected for global mean intensity.

Automated SICH prediction was implemented by a support vector machine (SVM), using a multilayer perceptron kernel, implemented within MATLAB R2012b. Given previous studies suggesting that SICH is associated with widespread brain features e.g. small-vessel ischemia, old strokes, or atrophy, (Gebel et al., 1998; Neumann-Haefelin et al., 2006; Sarikaya et al., 2011), in addition to inter-hemispheric asymmetry of parenchymal hypoattenuation (Dubey et al., 2001), we utilized the entire optimized CT image (single vector of 181,311 voxels per subject) as the feature-space input to our ‘automated’ SVM. Such a method is also more robust and user-independent than one requiring prior clinical, and/or radiologist judgements, regarding potentially relevant radiological features. Baseline NIHSS – which in our sample was the only significant non-radiological predictor of SICH – was incorporated by proportionately adjusting the contribution that each CT scan provided to the model. This was achieved by varying the soft margin parameter for each datapoint, inputted as a vector of NIHSS values into the ‘boxconstraint’ argument within the MATLAB svmtrain function. The resultant model generates a hyperplane across the entire image-feature space, relative to which future cases can be characterised. An alternative ‘manual’ SVM was constructed that inputted baseline NIHSS, together with radiological interpretations of acute ischemia extent and hyperdense MCA (Dharmasaroja and Dharmasaroja, 2012), rather than the images themselves.

To assess the predictive capabilities of SVMs, and established prognostic scores, we used k-fold cross-validation, by which we split the entire sample into 106 training (for SVM), and 10 test, subjects, over 1760 repetitions. The rationale for this was so that every test set was composed of 1 patient who developed SICH, and 9 who did not — thereby maximising the number of SICHs within each training set, at the same time as testing under an approximately realistic assumption of a 10% SICH frequency. Distances of each test image relative to the SVM hyperplane were estimated, from which classifications using one of ten evenly-spaced thresholds were made, allowing for AUC calculation. Additionally, for each test set of 10 subjects, the distances-to-hyperplane were ranked, from which the rank of the SICH target could be ascertained. This way we identified which of the 16 SICHs were successfully predicted by each model (i.e. assigned top rank out of 10).

Variations of the SVM model were also tested by eliminating baseline clinical severity as a soft-margin input; excluding brain base; and excluding the least-relevant cerebral hemisphere as judged clinically — i.e. from sensorimotor deficit laterality or aphasia. Lateralising features were present in all our patients; although whether patients were eventually diagnosed with brainstem–cerebellar stroke was not accounted for, given that practically such strokes cannot be reliably distinguished by clinical signs (Tao et al., 2012).

The study was ethically approved by the local Joint Research Compliance Office but did not require patient consent, using only retrospective, non-identifiable data.

## 3 Results

Characteristics of patients who developed SICH, versus those who did not, are displayed in Table 1. In our cohort the only significant predictors of SICH were baseline NIHSS; and CT evidence for acute ischemia (p < 0.05). For comparison with these standard predictors, the bottom two rows show support vector machine outputs for SICH and non-SICH groups, using raw CT images (‘automated’ SVM), or radiologist-derived scores (‘manual’ SVM), respectively (both in combination with baseline NIHSS).

Whilst both standardised prognostic scores and SVMs significantly discriminated SICH from non-SICH subjects, the relative classificatory performance of each type of prognostic system was assessed by calculating areas under receiver-operating characteristic curves (AUC) (Table 2, column 2). The AUC of the ‘automated’ SVM incorporating NIHSS and raw imaging data (0.744) was superior to that of the SEDAN and HAT scores, using either original or adapted versions of these scores (0.626–0.720; p < 0.01 for all). The ‘automated’ SVM was also superior to that of the ‘manual’ SVM that included baseline NIHSS and consensus radiologist interpretations (AUC: 0.671; 4 successful selections; p < 0.01).

We also assessed the relative performance of each classificatory system by seeing how many of the 16 SICHs, tested one at a time, each system could discriminate against 9 foils (this is equivalent to constraining each system to label only 10% of test items as SICH) (Table 2, columns 3–end). For this test, the automated SVM selected the correct subject 9 times (most commonly), as compared to 1–5 times using either original or adapted SEDAN and HAT scores, or ‘manual’ SVMs (p < 0.001). It is notable that the automated SVM predicted 3/4 cases where the SICH was remote from the original acutely ischemic territory, in comparison to none using the standard models.

Finally, the automated SVM as described showed inferior predictive performance when we removed baseline NIHSS from the model (AUC: 0.622); or by excluding the base image (AUC: 0.640); or by excluding the least-relevant hemisphere, as judged by clinical information (AUC: 0.557) (all comparisons with the whole-brain, NIHSS-adjusted SVM, p < 0.01).

## 4 Discussion

This is the first study to highlight the potential utility of imaging-based machine learning for predicting outcomes from stroke treatment. Not only have we shown that one such technique can offer automation — in place of error-prone radiology judgements (Wardlaw and Mielke, 2005), but also our results suggest that predictive performance may be enhanced over standard methods. Importantly, since our automated SVM was successful at SICH prediction using whole-brain as the input, rather than ad hoc feature combinations, and by assessing performance with cross-validation, our results are unlikely to have arisen by chance, or by data overfitting, despite a relatively small sample size. Furthermore, whilst the absolute improvement in AUC classificatory performance conferred by the ‘automated’ SVM was only ~ 2% greater than a conventional score-based system optimized for this dataset (and so not strictly cross-validated), the SVM was 10% better than the best prognostic system in its original, validated formulation; and moreover recognised twice as many SICHs when constrained to label only 10% of test items as hits, than any of the other methods. The probability of selecting, at random, 1 SICH out of 9 foils, in 9 out of 16 different tests, as the automated SVM achieved, is (from the binomial expansion) 5 × 10^− 6^.

A recent meta-analysis of SICH risk factors reported that at least 12 variables increase SICH likelihood, but their effect sizes are modest, emerging only from cohorts of thousands, and cannot in isolation identify patients at risk (Whiteley et al., 2012). This may explain why univariate analyses from our own relatively small dataset showed that only clinical severity and early ischemic CT changes were significant predictors of SICH. Prognostic systems – that integrate a range of risk factors into a single risk score – offer a practical way of stratifying risk. However, these have been shown in large validation cohorts to be far from optimal (Mazya et al., 2013), and achieved AUC values of only 63% in our cohort (using either), which might be because such methods assume linearity and independence between predictors. Machine learning by comparison does not make these assumptions, and may be the most suitable method when a very large number of factors (including imaging features) determine dichotomous outcomes, but where the mapping relationship is unknown or complex (Orrù et al., 2012).

One recent report showed that machine learning techniques can help with SICH prediction, but used radiologist interpretations (along with clinical variables), rather than images themselves (Dharmasaroja and Dharmasaroja, 2012). Replicating a similar technique with our dataset (‘manual’ SVM) did not improve predictive performance over existing prognostic scores. By contrast, the automated SVM that utilizes raw CT images, and clinical variables, was superior to the ‘manual’ SVM, and to standard prognostic scores (even after adapting these to include only significant predictors of our cohort). Similar to studies applying SVMs to MRI imaging in Alzheimer's disease (Klöppel et al., 2008), our results demonstrate that SVM techniques allow for effective classification where the number of features is far greater (here, ~ 180,000) than the number of training datapoints, unlike standard multivariate regression techniques.

Since the most successful imaging SVM we tested took whole-brain, rather than selected subparts, as its inputs, it seems likely that diffuse features, e.g. texture, morphometry, or low spatial-frequency changes, underlie the predictive effect we observe, rather than more focal features e.g. dense middle cerebral artery. This is supported by our finding that the SVM model was able to predict 3 out of 4 SICHs occurring remotely from the infarcted territory, as opposed to none predicted by conventional methods — implying that background brain features may be just as important as focal CT markers of acute ischemia. Certain background CT appearances, relating to small-vessel ischemia, old strokes and atrophy (Gebel et al., 1998; Neumann-Haefelin et al., 2006; Pantoni et al., 2014) are recognised predictors of SICH; as are several conditions that predispose to these radiological features, e.g. age, diabetes, renal failure (Whiteley et al., 2012). Although the risk sizes associated with each of these factors are modest, and whilst we did not find an association between SICH and hemispheric white matter lesion load, it is possible that crude, human estimates of overall white-matter load (Wahlund et al., 2001), are less relevant to haemorrhage risk than spatial patterns of hypoattenuation across the whole brain, that the SVM model takes as inputs. The SVM model may also appreciate patterns of cerebral atrophy related to amyloid angiopathy (Erten-Lyons et al., 2013) — itself a predisposing factor for intracerebral haemorrhage.

A further possible explanation for the SVM's predictive ability is that it is sensitive to the extent of *focal*, acute ischemic changes (territorial hypoattenuation, or grey–white matter effacement), that are associated with SICH (Whiteley et al., 2012), as was also found in our sample from a univariate analysis. The fact that the SVM taking a whole-brain input was more successful than inputting only the clinically-relevant brain part, or cerebrum only, may be because sensitivity to acute ischemic changes is optimized by comparing feature patterns *between* hemispheres (Dubey et al., 2001), or *between* posterior fossa and hemispheres; or because the possibility that some strokes were in the posterior circulation was not taken into account by analyses restricted to cerebral hemispheres (given the practical scenario in which only clinical information and early unenhanced CT are available, which are only poorly able to distinguish arterial territory (Tao et al., 2012)). However, we also found that the SVM's performance improved by adjusting for clinical severity — similar to SEDAN and HAT scores, that combine clinical severity with acute radiological features. This suggests that acute ischemic changes evident on unenhanced CT are less accurate at reflecting acute ischemia volume – a critical determinant of SICH (Campbell et al., 2012) – as when clinical severity is factored in (Fink et al., 2002). Thus our SVM model of SICH risk may reflect an interaction between background brain features and acute ischemia volume, the latter is at least partly estimable by clinical assessment.

Whilst our results indicate that machine learning may help predict SICH over existing prognostic schemes, we recognise that our study has several limitations. Firstly, only a relatively small number of SICH cases were available (representing ~ 5% treated cases from a single stroke centre), making it difficult to know whether an SVM model similar to the one used here could generalize to a larger number of cases, treated in different centres, using different CT protocols etc. Future studies should include all thrombolysed patients, including non-SICH subjects whose NIHSS score did not improve with tPA, or asymptomatic intracranial haemorrhages, both of which we excluded from this exploratory study. It would also be interesting to test predictive models on patients who were potentially-suitable for thrombolysis but not treated with thrombolysis because of clinicians' judgements about SICH risk. Acquiring an unbiased dataset may be best achieved prospectively, although incorporation of real-world, noisy data, e.g. by using NIHSS evaluations from a range of specialists (as here), is desirable in developing a predictive model for use in other real-world contexts.

A second problem, and one that will most likely apply to all future studies of SICH prediction, is that such datasets will be highly imbalanced towards cases without SICH. Imbalanced designs tend to bias models towards the majority case, which in this context, may exacerbate false-negative errors, but which can be mitigated by various adaptive methods (Mazurowski et al., 2008). To this end, we lessened the imbalance by downsampling from the non-SICH group whilst ensuring that those analysed did not differ significantly from those not.

A third set of challenges, that we have not explored, relates to the selection of image-space features, which may improve classification. However, with relatively small datasets, as here, there is a danger that ad hoc feature optimization overfits the data, identifying features by chance. A further issue, given the highly-variable, territorial nature of stroke is that the anatomical features selected may need to be adapted to individual cases — unlike the case for many existing neuroimaging machine learning applications e.g. Alzheimer's disease. For example, based upon established radiological predictors of SICH, relevant features may be those related to unilateral middle cerebral artery density, or acute ischemic changes (Whiteley et al., 2012). However, this then becomes problematic because of the need for interpretation, e.g. is a right hemiparesis due to infarction of the left hemisphere or brainstem? (Tao et al., 2012); and extra image processing, e.g. individualised demarcation of the middle cerebral artery, or its supplied territory. Recent automated methods to identify acute ischemic changes or vessel hyperdensity (Takahashi et al., 2012, 2014) may be useful in this regard. It is also likely that advanced imaging techniques e.g. CT angiogram/perfusion or MRI, which are increasingly used in clinical practice, and are more sensitive than non-enhanced-CT to haemorrhage-associated features e.g. microbleeds (Charidimou et al., 2013), or infarct volume (Campbell et al., 2012), may provide superior information to machine learning-based models, but raise the challenge of how multimodal data can be optimally combined for prediction (Gray et al., 2013).

A final consideration is that the image-processing steps required for case prognostication here were relatively inefficient (scans had to be exported, and two sections had to be joined), and timely (~ 30 minute processing per scan). Clearly, for such a method to have any practical usefulness these steps would need to be made more rapid and automated, and ideally, implemented on a single scanner PC.

In conclusion, our study shows that machine learning applied to unenhanced CT can distinguish patients destined to suffer thrombolysis-associated SICH, from those who will respond well to thrombolysis; and moreover, perform this with higher accuracy than conventional, radiology-based methods. Larger studies are justified to explore such techniques in patients with all types of thrombolysis outcome; and for other important stroke-prognostic questions, e.g. predicting successful responses to treatment, and long-term functional recovery.

## Conflicts of interest

None.

## Figures and Tables

**Table 1:** Clinical and radiological characteristics of SICH and non-SICH groups.

Variable	SICH	No SICH	Odds ratio	p-Value	No SICH (excluded)
Gender/% males	63	49	1.74 (0.59–5.14)	0.320	51
Age/yrs	75.1 (69.3–80.9)	73.2 (70.7–75.7)	1.01 (0.97–1.05)	0.572	70.6 (68.3–72.9)
Treatment delay/min	136 (112–159)	146 (134–158)	1.00 (0.99–1.01)	0.509	152 (139–166)
Baseline NIHSS/42	15.3 (12.5–18.0)	12.3 (11.2–13.3)	1.10 (1.00–1.21)	0.048*	10.2 (9.1–11.3)
Systolic blood pressure/mm Hg	161 (152–170)	162 (156–167)	1.00 (0.98–1.02)	0.949	155 (151–159)
Glucose/mmol/l	7.09 (6.3–7.9)	7.55 (7.04–8.05)	0.92 (0.71–1.18)	0.490	7.26 (6.88–7.65)
INR	1.08 (1.04–1.11)	1.31 (1.05–1.57)	0.14 (0.00–28.5)	0.329	1.16 (1.01–1.32)
Platelets/× 10^9^/l	234 (193–276)	238 (221–254)	1.00 (0.99–1.01)	0.880	253 (242–265)
Anti-thrombotic therapy/%	75	62	1.84 (0.55–6.11)	0.320	51
CT — acute ischemia/%	63	27	3.31 (1.12–9.74)	0.008**	24
CT — acute ischemia > 1/3 MCA territory/%	31	6	5.22 (1.29–21.2)	0.004**	3
CT — hyperdense MCA sign/%	38	17	4.41 (1.42–13.6)	0.064	19
CT — white matter Fazekas score/3	0.81 (0.46–1.17)	1.13 (0.95–1.31)	0.64 (0.33–1.24)	0.185	1.14 (1.01–1.26)
SEDAN score/6	2.50 (2.97–2.03)	2.05 (1.82–2.28)	1.43 (0.90–2.30)	0.146	1.66 (1.46–1.85)
HAT score/5	1.63 (0.96–2.29)	1.03 (0.82–1.24)	1.49 (0.95–2.32)	0.053	0.75 (0.60–0.89)
SEDAN score — NIHSS, CT only/3	1.88 (1.44–2.31)	1.10 (0.91–1.29)	2.23 (1.28–3.89)	0.006**	0.82 (0.65–0.95)
HAT score — NIHSS, CT only/5	1.63 (0.96–2.29)	0.95 (0.75–1.15)	1.60 (1.02–2.52)	0.027*	0.64 (0.50–0.78)
‘Automated’ SVM/distance from hyperplane (arbitrary units)	− 3.25 (− 6.47–−0.03)	3.25 (2.31–4.19)	1.28 (1.12–1.45)	< 0.0001**	n/a
‘Manual’ SVM/distance from hyperplane (arbitrary units)	− 3.55 (− 8.45–1.34)	3.55 (1.81–5.30)	1.08 (1.02–1.14)	0.008**	n/a

Mean (95% confidence intervals) quoted. Odds ratio and p-value relate to univariate logistic regression analyses comparing SICH with non-SICH. SICH: Symptomatic Intracranial Haemorrhage; NIHSS: National Institute of Health Stroke Scale; INR: International Normalised Ratio. SEDAN score predictors: glucose (2 points), baseline NIHSS, CT acute ischemia, hyperdense MCA sign, and age (1 point each); HAT score predictors: glucose (1 point), CT acute ischemia > or < 1/3 MCA territory, and baseline NIHSS (2 points each). ‘Adapted’ HAT and SEDAN Scores (last two rows) included only NIHSS and radiological components of their original versions, with identical weightings for these. ‘Automated’ and ‘Manual’ SVMs represent subjects' predicted output values from support vector machines using raw images, or radiological scores (acute ischemia, hyperdense MCA sign), respectively, *< 0.05; **< 0.01. Final column characterises non-SICH subjects from the total cohort that were not analysed; this group did not differ significantly from the analysed group in any of the measured variables.

**Table 2:**
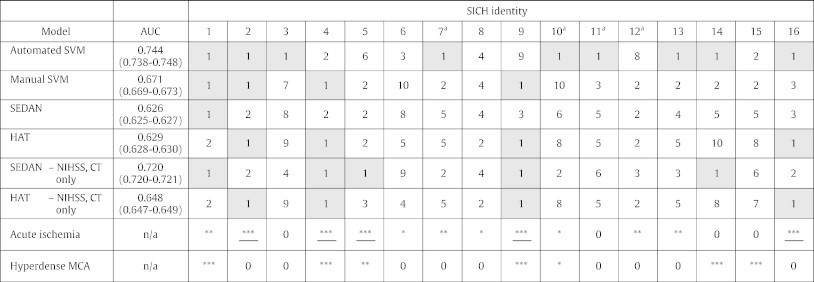
Predictive performance of automated SVM compared to other prognostic methods.

SVM: Support vector machines, using either raw images (‘automated’) or radiologist-derived reports of acute ischemia and hyperdense MCA (‘manual’). AUC: area under receiver operating characteristic curve (95% confidence intervals). SICH identity: For each SICH, the commonest ranking accorded by each model to the SICH subject is reported, across 110 tests, comprising 1 patient who develops SICH, and 9 who recover. Coding: ‘1’ represents most likely to develop SICH, and ‘10’ least likely. A bold '1' (shaded box) indicates that the specified model correctly predicted the specified SICH, in terms of placing it most likely out of 9 alternatives. Final two rows indicate the number of radiologists (0–3 represented as *) reporting acute ischemia (underlined if majority judged to be > 1/3 MCA territory), or MCA hyperdensity. ^a^These subjects' haemorrhages were judged to be remote from the acute ischemic territory.
